# Evaluation of an Integrated Teaching Approach in Physiology, Anatomy, and Biochemistry for Phase I MBBS Students Using the Kirkpatrick Model

**DOI:** 10.7759/cureus.88879

**Published:** 2025-07-28

**Authors:** Shubhanshu Gupta, Sameer Sathe, Kalpana Arya, Bikramjeet Mitra, Anil Mangeshkar

**Affiliations:** 1 Community Medicine, Government Medical College, Datia, IND; 2 Anatomy, Government Medical College, Datia, IND; 3 Forensic Medicine and Toxicology, Government Medical College, Datia, IND

**Keywords:** didactic, integrated teaching, kirkpatrick’s evaluation model, likert scale, teaching-learning method

## Abstract

Introduction

Integrated teaching methods are increasingly emphasized in medical education to bridge the gap between basic and clinical sciences, leading to early clinical reasoning and better learning outcomes. The study aims to evaluate the effectiveness of an integrated teaching-learning approach for Phase I MBBS students using the Kirkpatrick evaluation model.

Methods

This educational interventional study was conducted from September 2023 to August 2024 at Government Medical College, Datia, involving 150 Phase I MBBS students. Three core topics, Cardiac Cycle (Physiology), Heart (Anatomy), and Lipid Metabolism (Biochemistry), were covered through didactic lectures under an integrated teaching program. A pre-test and post-test design was employed to assess knowledge gain. The intervention was evaluated at three levels of the Kirkpatrick model: reaction (student satisfaction), learning (knowledge improvement), and behavior (student perceptions regarding program organization). Student feedback and faculty feedback were collected using non-validated structured questionnaires. Data were analyzed using descriptive statistics and paired t-tests in Jamovi v2.3.28, with p < 0.05 considered statistically significant.

Results

Participants were 52% men and 48% women, with a mean age of 19.2 ± 1.1 years. Student feedback revealed high satisfaction, with the majority rating the sessions positively. Significant improvements were observed in post-test scores across all subjects (Anatomy: p = 0.036; Physiology: p = 0.001; Biochemistry: p = 0.004). Organizational aspects of the program were rated highly.

Conclusion

The integrated teaching approach effectively enhanced student satisfaction and learning outcomes. The findings support the inclusion of integrated teaching as a regular strategy in the medical curriculum to improve foundational learning.

## Introduction

Medical education has transformed significantly in recent years, emphasizing the integration of foundational sciences with clinical applications to enhance learning outcomes and clinical competence. Integrated teaching methods bridge the gap between basic and clinical subjects, promoting contextual learning and early clinical reasoning during the initial (Phase I) years of undergraduate medical training.

Integrated teaching is a learner-centered approach that merges overlapping concepts across disciplines, horizontally within a phase and vertically across phases, to enhance clinical relevance, reduce redundancy, and foster holistic understanding. It aligns teaching temporally and thematically to promote contextual, competency-based learning in undergraduate medical education [1].

The Kirkpatrick model remains one of the most widely adopted frameworks for assessing the effectiveness of educational interventions, and the four-level model evaluates learners’ reactions, learning, behavior, and results, providing a systematic approach to assessing the effectiveness of both formal and informal training programs [2]. The model focuses on immediate satisfaction and knowledge acquisition, as well as the application of skills in real-life settings and their broader impact on organizational outcomes.

Kulkarni et al. demonstrated that the use of multiple case scenarios in integrated teaching significantly enhanced conceptual clarity, motivation, and critical thinking among first-year medical students, with over 95% of students expressing a positive impact on their learning experience [3]. A study in Bangladesh reported a significant improvement in academic performance and student satisfaction following integrated sessions, although it highlighted the need for improved clinical correlation and simulation elements [4]. Sarkar et al. found that a blended learning model combining integrated content delivery with online tools enhanced student engagement, promoted active learning, and improved self-directed learning readiness among Phase I MBBS students [5].

Ragsdale et al. advocated for using the Kirkpatrick model in evaluating undergraduate clinical education programs, emphasizing that evaluations should encompass baseline, process, and outcome measures, particularly regarding improved patient care outcomes [6]. Johnson et al. proposed an expanded evaluation framework adapted from the New World Kirkpatrick Model, incorporating additional levels to assess the broader impacts of public and population health curricula on healthcare systems and population health [7]. This reflects the increasing recognition of the model’s versatility in capturing both immediate and distal effects of educational programs.

El Nsouli et al. found that most studies evaluating interprofessional simulation activities among pharmacy students focused on the initial two Kirkpatrick levels (reaction and learning), with limited attention to behavioral change and long-term outcomes, highlighting areas for improvement in evaluation practices [8]. Menezes et al. reviewed educational interventions targeting empathy and compassion in medical students and noted that most studies achieved improvements in knowledge and behavior, although sustained changes required well-integrated, longitudinal interventions [9].

This study aims to apply the Kirkpatrick evaluation model; Levels 1 (reaction), 2 (learning), and 3 (behavior) were evaluated systematically to assess the effectiveness of an integrated teaching method for Phase I MBBS students.

## Materials and methods

This educational interventional study was conducted over a period of one year in three departments of Phase I MBBS, Anatomy, Physiology, and Biochemistry, of Government Medical College, Datia, using the Kirkpatrick evaluation model. A total of 150 students participated in the study.

Study area

The study was conducted in the Departments of Anatomy, Physiology, and Biochemistry of Government Medical College, Datia, Madhya Pradesh, India.

Study duration

The study was conducted from September 2023 to August 2024 over one academic year.

Inclusion and exclusion criteria

Phase I MBBS students willing to participate in the study and students who provided written informed consent were included in the study. Only participants who attended the integrated session and completed both tests were included in the study.

Students absent during either the pre-test, teaching sessions, or post-test and who declined to participate or did not provide consent were excluded from the study.

Methodology

An interdepartmental coordination committee was established, comprising a professor from each of the three basic science departments, the head of each department, and faculty members from the medical education unit. This committee collaboratively designed, coordinated, implemented, and evaluated an integrated teaching program.

Three topics selected for the integrated teaching sessions were the Cardiac Cycle (Physiology), Heart (Anatomy), and Lipid Metabolism (Biochemistry). Topics were selected to represent horizontal integration across pre-clinical subjects and to get vertical relevance by introducing clinically important biochemical concepts such as lipid metabolism in the context of cardiovascular physiology, helping students understand their practical application in medical practice.

The intervention involved consecutive, thematically aligned didactic lectures delivered by faculty from various disciplines in a coordinated format and was pre-planned, unlike routine sessions for intradisciplinary and contextual learning. A pre-test was administered three days prior to the lecture sessions to assess baseline knowledge. After the completion of the teaching sessions, a post-test was conducted three days later to evaluate knowledge gain. The same 11-item questionnaire was administered before and after the intervention.

**Figure 1:**
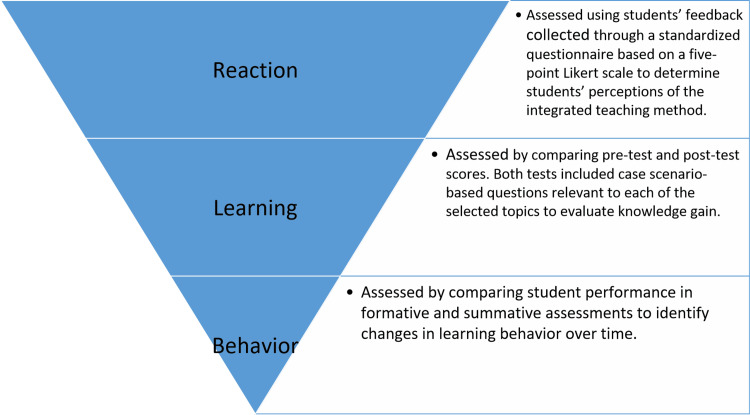
The Kirkpatrick evaluation model Image credits: The authors

The program’s effectiveness was evaluated using three levels of the Kirkpatrick model: Level 1 (reaction) to assess student satisfaction and perception, Level 2 (learning) to measure knowledge improvement through pre- and post-test scores, and Level 3 (behavior) to evaluate students’ perceptions of the organization and relevance of the program. Level 4 (results) was not evaluated due to the study’s limited temporal scope and lack of long-term outcome measures. The overall study design and the evaluation process based on the Kirkpatrick model are summarized in Figure 1.

Student feedback was collected using a structured, non-validated questionnaire developed by our team to assess the students, and no other validated tools were used. The questionnaire was used to collect data on participants' perceptions of the teaching-learning (TL) method. It included sections on sociodemographic details, such as age and gender, along with items assessing prior exposure to the TL method. It consists of Likert scale questions evaluating various aspects of the learning experience using the Kirkpatrick model of evaluation. The detailed questionnaire is provided in the appendix. The specific questions used for student feedback are listed in Table 1.

**Table 1:** Student feedback questionnaire with questions TL: teaching-learning method

Serial no.	Question
1	I am interested in participating in this TL method
2	This TL method is stimulating
3	It helped to develop my problem-solving skills in future practice
4	The teaching time is put to good use
5	The teacher overemphasizes factual learning
6	The teacher had good interaction with us
7	This TL method is useful for me
8	I feel confident of passing with more problem-solving sessions
9	Enjoyment in the sessions outweighs the stress of studying
10	I can clear doubts with the teacher
11	This TL method motivated me as a lifelong learner

Statistical analysis

Statistical analysis was performed using Jamovi v2.3.28 (retrieved from https://www.jamovi.org). Students’ feedback was analyzed using a five-point Likert scale. The results are presented as numbers and percentages. Pre-test and post-test scores were compared using a paired t-test. Responses on the five-point Likert scale were categorized as follows: scores of 1-2 were classified as “disagree,” 3 as “neutral,” and 4-5 as “agree.” A p-value of <0.05 was considered statistically significant.

Ethical considerations

The study was approved by the Institutional Ethics Committee of Biomedical Research and Human Participants (IEC Approval No: 125/CM/GMC/IECBMHR/2023, Date: April 15, 2023). Anonymity was maintained, and no information regarding the participants was disclosed.

## Results

A total of 150 Phase I MBBS students participated in the study, comprising 78 men (52%) and 72 women (48%). The age of participants was found to be normally distributed (p > 0.05) and ranged from 18 to 25 years, with a mean age of 19.2 ± 1.1 years.

Students’ perception of teaching and learning through integrated teaching

The majority of students responded positively to the integrated TL method. Over 85% agreed it was interesting (Q1), useful (Q7), and made effective use of time (Q4). Around 68% believed it enhanced problem-solving skills (Q3) and built confidence (Q8). However, only 10% agreed that the teacher maintained good interaction (Q6), and just 9% felt doubts were effectively cleared (Q10), indicating communication gaps. Most disagreed that the teacher overemphasized factual learning (Q5), supporting a more conceptual approach. Overall, students viewed the method as motivating (Q11), though improvements in teacher engagement and interactive support are needed (Table 2).

**Table 2:** Students’ perception of teaching and learning via integrated teaching (N = 150) TL: teaching-learning Response categories: poor/satisfactory (Likert 1 & 2), neutral (Likert 3), and good/better/excellent (Likert 4 & 5).

Question	Likert scale	Strongly agree (%)	Agree (%)	Neutral (%)	Disagree (%)	Strongly disagree (%)
Q1	Interested in participating in this TL method	33	58	15	4	0
Q2	This TL method is stimulating	17	49	25	8	1
Q3	It helped develop problem-solving skills for future practice	19	49	27	0	2
Q4	Teaching time was effectively used	21	64	12	3	0
Q5	The teacher overemphasized factual learning	0	4	16	38	42
Q6	The teacher maintained good interaction	2	8	24	39	27
Q7	This TL method is useful for me	27	57	13	3	0
Q8	I feel confident of passing with more problem-solving sessions	30	38	30	1	1
Q9	Enjoyment outweighed study stress	5	52	33	10	0
Q10	I was able to clear doubts with the teacher	1	8	41	36	14
Q11	This TL method motivated me as a lifelong learner	17	54	22	3	4

Comparison of pre-test and post-test scores

In Anatomy, the mean post-test score increased to 8.50 from a pre-test mean of 2.86 (p = 0.036), indicating a statistically significant improvement. Similarly, Physiology scores rose from 2.95 to 7.46 (p = 0.001), and Biochemistry scores improved from 2.75 to 8.26 (p = 0.004). These results suggest that the TL intervention had a significant positive impact on student performance across all subjects (Table 3).

**Table 3:** Comparison of pre-test and post-test scores across sessions (N = 150) *p < 0.05; **p < 0.01. A paired t-test was used.

Subject	Test type	Mean ± SD	p-value
Anatomy	Pre-test	2.86 ± 1.30	0.036*
	Post-test	8.50 ± 1.20	
Physiology	Pre-test	2.95 ± 1.22	0.001**
	Post-test	7.46 ± 1.85	
Biochemistry	Pre-test	2.75 ± 1.15	0.004**
	Post-test	8.26 ± 1.80	

Students’ perception of the organization of the integrated teaching program

The majority of participants reported positive perceptions regarding the organization of integrated teaching sessions. Agreement was highest for interdepartmental coordination (90%) and availability of adequate resources (89%), followed by appropriate scheduling (82%) and timely communication (80%). Neutral responses remained moderate (14%-19%) across items. Disagreement was minimal for coordination (8%) but notably higher for overall satisfaction (36%), indicating room for improvement (Table 4).

**Table 4:** Students’ perception of organization of integrated teaching program (N = 150) Responses were recorded on a five-point Likert scale: 1: strongly disagree; 2: disagree; 3: neutral; 4: agree; 5: strongly agree.

Question	Likert scale	Strongly agree (%)	Agree (%)	Neutral (%)	Disagree (%)	Strongly disagree (%)
Q1	Adequate scheduling and planning	16	82	18	14	14
Q2	Timely communication regarding sessions	17	83	19	18	11
Q3	Adequate coordination between departments	19	87	19	17	8
Q4	Sufficient resources for integrated sessions	21	88	14	13	10
Q5	Time allocation was appropriate	16	84	14	18	12
Q6	Overall satisfaction with the organization	12	83	18	19	17

## Discussion

The present study evaluated the effectiveness of an integrated TL approach for Phase I MBBS students using the Kirkpatrick evaluation framework, with promising results observed at the reaction, learning, and behavior levels.

Our study highlights that student perceptions had high levels of satisfaction with the integrated TL method, with more students rating the sessions as good, better, or excellent, particularly in terms of engagement, usefulness, and motivation for lifelong learning. Debnath et al. emphasized the value of two-way feedback mechanisms in improving teaching methods and fostering student engagement, with nearly all students in their study endorsing such strategies for teaching improvement [10]. Constantinou and Wijnen-Meijer advocated for a comprehensive, multi-faceted evaluation system that extends beyond student satisfaction alone to improve teaching effectiveness [11].

Knowledge acquisition significantly improved post-intervention across all subjects in the current study, as evidenced by statistically significant increases in post-test scores. This is consistent with studies that have shown the effectiveness of integrated teaching approaches. Kaikaew et al. reported that two-dimensional integrative teaching approaches improved students’ understanding, critical thinking, teamwork, and academic performance [12]. Adhikari Yadav et al. found that the Clinical Presentation curriculum enhanced students’ knowledge retention and was preferred by both students and faculty over traditional teaching methods [13]. Our results show that integrating basic and clinical sciences can lead to meaningful improvements in student learning outcomes in foundational subjects.

The organization and delivery of the integrated teaching program were also rated highly by students, indicating satisfaction with regard to scheduling, coordination, and availability of resources. Allana et al., in their evaluation of an integrated bioethics curriculum, highlighted the significance of structured planning, role modeling, and practical learning experiences in achieving educational objectives [14].

Allen et al. argued that while such models are useful for measuring outcomes, they often overlook the underlying processes and contextual factors that contribute to those outcomes [15]. Alexandraki et al. highlighted that evaluations of faculty development programs often neglect rigorous outcome measures, suggesting a broader need for systematic and process-oriented evaluations in medical education [16]. Our study, while effective in demonstrating short-term learning gains and positive reactions, does not capture longer-term outcomes, such as sustained knowledge retention, clinical application, or patient care improvements for future research.

Kelly et al. noted that emerging teaching modalities, such as podcasts, have been found to be effective adjuncts in medical education, offering flexibility and knowledge retention comparable to traditional methods [17]. Such innovations may complement the integrated curriculum with the TL method to further enhance learning efficiency.

A study applied this model to evaluate an English as a Medium of Instruction (EMI) training program for medical faculty, demonstrating substantial improvements in participants’ teaching competence and confidence, along with high satisfaction levels, indicating that the program effectively enhanced both individual capacity and learner-centered pedagogy [18].

Simulation-based medical education (SBME) has also been shown to enhance students’ theoretical knowledge in hematology. A 2021 study demonstrated that SBME significantly outperformed traditional lecture-based methods, with students valuing simulation sessions as impactful learning experiences [19].

Limitation

This study was conducted at a single institution with a modest sample size of 150 students, which may limit the generalizability of its findings to other medical colleges or diverse educational settings. It assessed short-term learning outcomes through pre- and post-test scores but did not evaluate long-term knowledge retention or impact on clinical performance. The study primarily addressed the first three levels of the Kirkpatrick model, reaction (Level 1), learning (Level 2), and behavior (Level 3), while omitting Level 4, which examines long-term outcomes on patient care and institutional performance. Although Level 3 focuses on behavioral change, we used self-assessment as a proxy, which may not substitute for performance-based evaluations. Questions 9-11 reflected students’ intention to apply integrated knowledge, but this may not confirm actual behavior change and should be interpreted with caution. The use of self-reported feedback introduces potential response bias. The non-validated questionnaire limits the reliability of the findings. The study evaluated only didactic sessions, excluding active learning methods such as case-based discussions, problem-based learning, and clinical applications, which may affect the broader applicability of the integrated teaching approach. The intervention was restricted to pre-clinical subjects (Physiology, Anatomy, and Biochemistry) and did not include clinical sciences.

## Conclusions

Integrated teaching for Phase I MBBS students was effectively evaluated using the Kirkpatrick evaluation model, which provided a comprehensive assessment of student satisfaction, learning outcomes, and application of knowledge. The evaluation clearly demonstrated that the integrated teaching method achieved its intended goal. Based on these findings, integrated teaching can be successfully implemented as a routine TL strategy in the medical curriculum. More research with a larger sample size is needed to generalize the findings for a better assessment of the integrated teaching method.

## Figures and Tables

**Figure 2:**
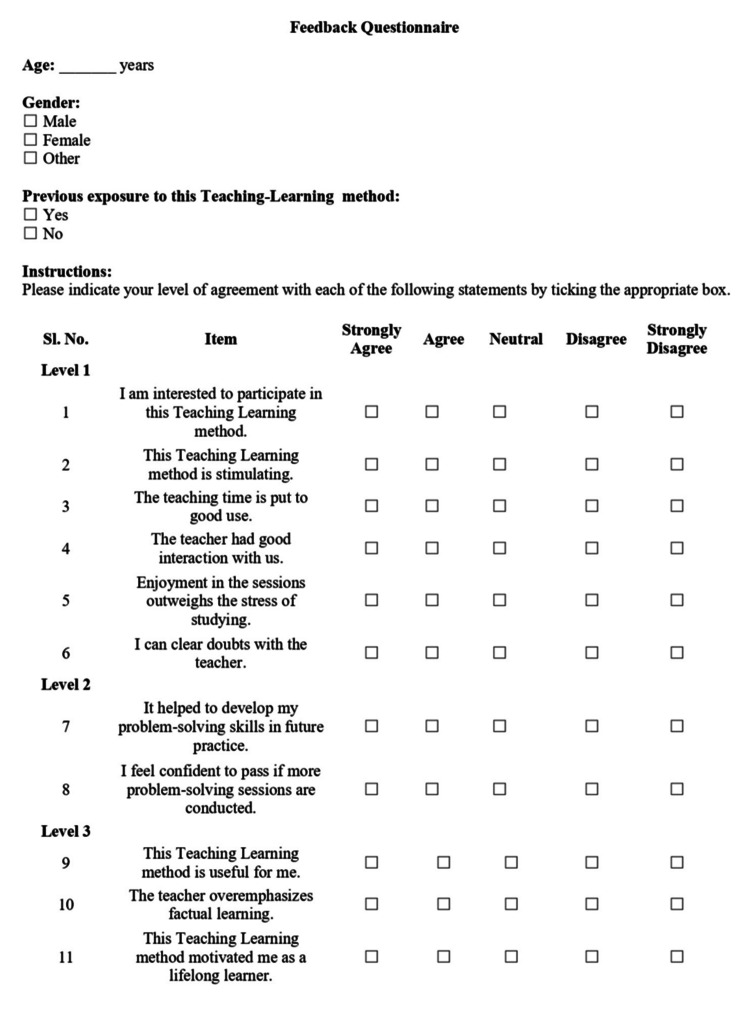
Structured feedback questionnaire on the teaching-learning method based on different levels of the Kirkpatrick model
